# Rational Design of Metal–Organic Frameworks for Electroreduction of CO_2_ to Hydrocarbons and Carbon Oxygenates

**DOI:** 10.1021/acscentsci.2c01083

**Published:** 2022-10-25

**Authors:** Hao-Lin Zhu, Jia-Run Huang, Pei-Qin Liao, Xiao-Ming Chen

**Affiliations:** MOE Key Laboratory of Bioinorganic and Synthetic Chemistry, School of Chemistry, Sun Yat-Sen University, Guangzhou 510275, China

## Abstract

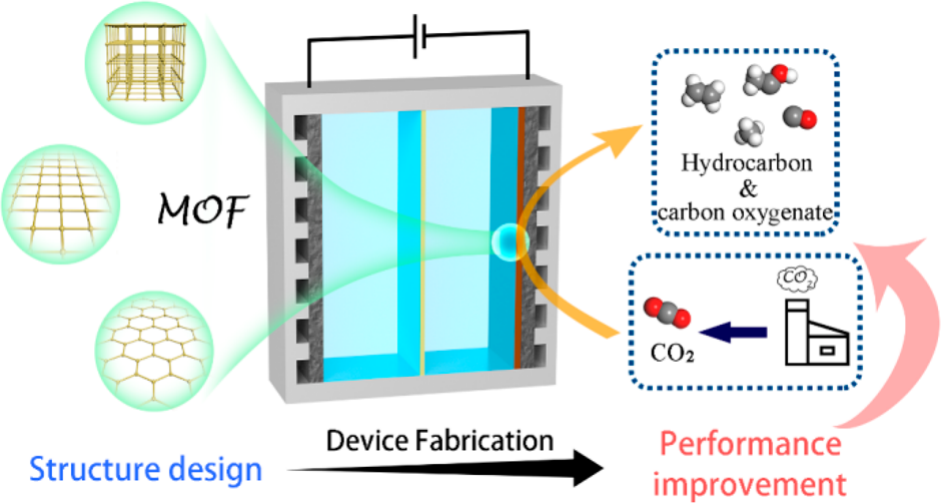

Since CO_2_ can be reutilized by using renewable electricity in form of product diversity, electrochemical CO_2_ reduction (ECR) is expected to be a burgeoning strategy to tackle environmental problems and the energy crisis. Nevertheless, owing to the limited selectivity and reaction efficiency for a single component product, ECR is still far from a large-scale application. Therefore, designing high performance electrocatalysts is the key objective in CO_2_ conversion and utilization. Unlike most other types of electrocatalysts, metal–organic frameworks (MOFs) have clear, designable, and tunable catalytic active sites and chemical microenvironments, which are highly conducive to establish a clear structure–performance relationship and guide the further design of high-performance electrocatalysts. This Outlook concisely and critically discusses the rational design strategies of MOF catalysts for ECR in terms of reaction selectivity, current density, and catalyst stability, and outlines the prospects for the development of MOF electrocatalysts and industrial applications. In the future, more efforts should be devoted to designing MOF structures with high stability and electronic conductivity besides high activity and selectivity, as well as to develop efficient electrolytic devices suitable for MOF catalysts.

## Abstract 2

As promising catalysts of CO_2_ electroreduction, metal−organic frameworks are expected to exhibit improved catalytic performance through the rational design of active site structures and their microenvironments, as well as electrolytic devices.

Rapid development of industry requires increasing consumption of fossil fuels, which has led to a serious rise of the carbon dioxide (CO_2_) content in the atmosphere and an escalation of the energy crisis.^1^ A dramatic rise of atmospheric CO_2_ concentration from 315.7 ppm in March 1958 to 418.9 ppm in July 2022 was observed in Hawaii,^2^ confirming the global greenhouse effect as well as ocean heat uptake.^3,4^ Hence, the capture and conversion of CO_2_ is regarded as an urgent task in this century.^1^

The renewable electricity powered electrochemical CO_2_ reduction reaction (ECR) is a prospective approach for CO_2_ utilization and energy storage. In mild reaction conditions, CO_2_ can be effectively converted into various value-added hydrocarbons, alcohols, and organic acid products through the ECR reaction.^5^ Up until now, many metal-based electrocatalysts including metal nanoparticles, single-atom materials, and molecular/metal complexes exhibit state-of-the-art electrochemical performances toward ECR.^6^ Despite the commendable progress, ECR usually exhibits enhanced performance in high alkaline electrolytes (*e*.*g.*, 0.1 M KOH, 0.5 M KOH, and 1 M KOH aqueous solution),^7^ yet possibly causes CO_2_ wastage and carbonate deposition. Therefore, further improvement of ECR performance requires the precise design of catalysts. However, the preparation of metal bulks, nanoparticles, and single-atom catalysts always requires a special synthetic process with harsh conditions, and the insufficient clarity of the active sites is adverse to the in-depth comprehension of the reaction mechanism, which is critical to further optimization of the catalysts toward practical applications. Therefore, new types of electrocatalysts should be developed to reveal the thorough structure–performance relationship and achieve the requirement of industrial applications. Metal–organic frameworks (MOFs) and relevant molecule-based porous materials are a nice platform for heterogeneous ECR investigations due to their large surface areas and tunable framework structures.^8−18^ More importantly, the periodic and well-defined catalytic sites in MOFs for substrate interactions can be straightforwardly detected and studied at atomic and/or molecular levels by using experimental techniques and theoretical calculations, promoting the study of the structure–performance relationship and reaction mechanism.^10^ MOFs were used as catalysts for ECR in 2012 for the first time.^19,20^ Up to now, many MOFs, especially metal-azolate frameworks (MAFs),^21−23^ have been proven to be robust and highly efficient for the ECR process (Figure 1). Despite the many advantages showcased by MOF electrocatalysts, their industrial applications are still restricted by several non-negligible shortcomings such as the low current density and stability. Therefore, more studies and discussions on MOF catalysts for ECR are anticipated. Although a number of reviews^8−12,24^ have discussed the applications of MOFs in ECR, the regulation of MOFs on the selectivity, current density, and stability, especially from the perspective of coordination chemistry, has not been discussed. This Outlook aims to concisely review the very recent progress on the MOF-based electrocatalysts for ECR and outline critical insights into the structure–performance relationship and performance adjustment. We will also give forward-looking viewpoints on the industrial potential of MOF electrocatalysts.

**Figure 1:**
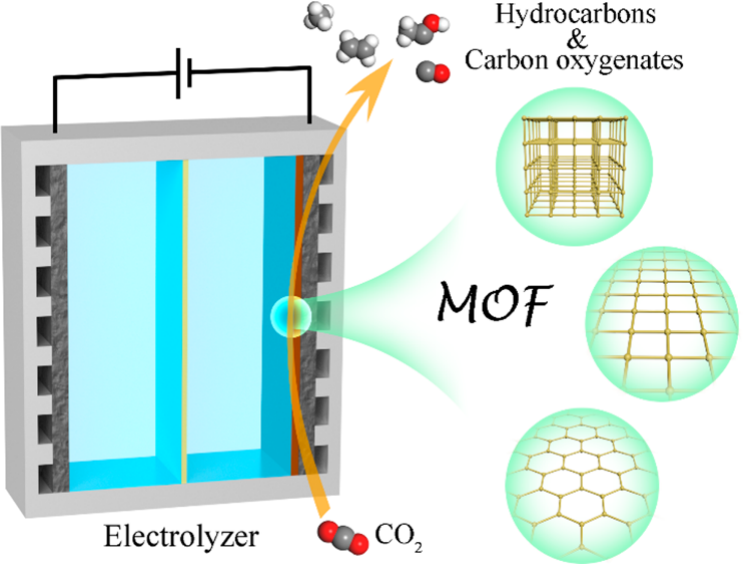
MOFs as highly efficient catalysts for ECR.

## Selectivity Control

**Figure 2:**
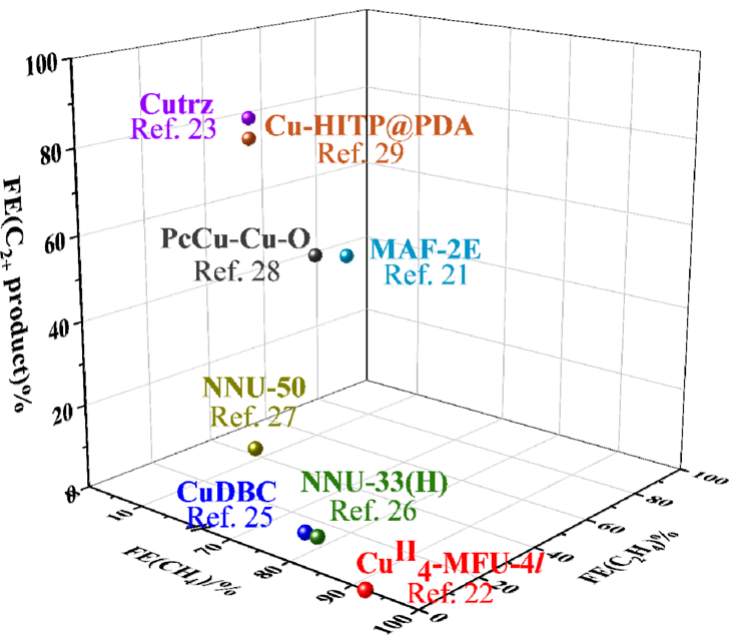
Comparison of FEs for yielding CH_4_, and C_2_H_4_, and C_2+_ products by using different Cu-MOFs for ECR.

**Figure 3:**
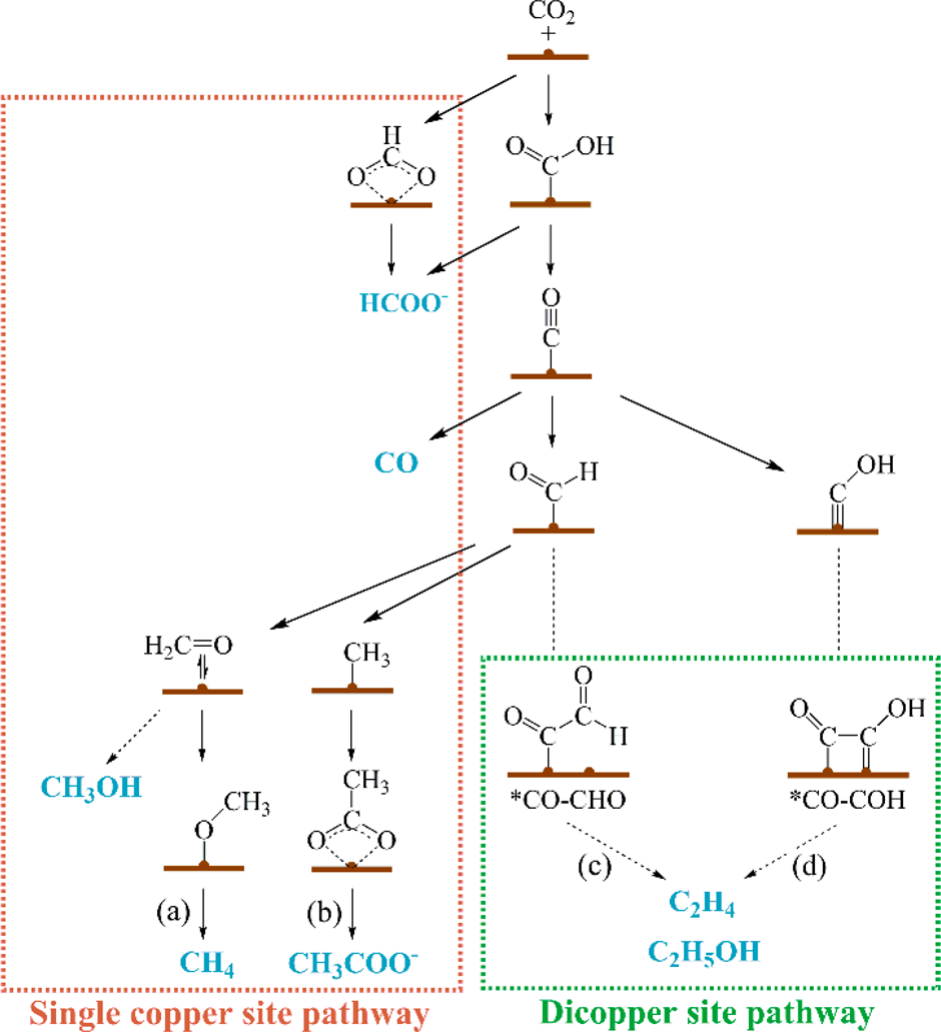
Electroreduction pathways of CO_2_ to the most common products catalyzed by MOFs and other coordination compounds.

Since various products could be yielded from ECR and the complexity of products limits the improvement of energy usage and leads to non-negligible product separation and enrichment issues, the catalytic reaction should be controlled to yield the targeted product as the sole or at least the main product. As MOFs have the advantages of designable frameworks and tailorable microenvironments, compared with other types of catalysts, using MOFs as electrocatalysts can easily adjust the product composition. To date, the highest Faradaic efficiency (FE) for yielding CH_4_, C_2_H_4_, and C_2+_ products based on MOF electrocatalysts reaches >80%,^22,25,26^ >50%,^23,28,29^ and 80%,^23^ respectively (Figure 2 and Table 1). Previous reports revealed that the electrocatalysts with Au,^30,31^ Ag,^32^ Co,^33−35^ or Ni^36−40^ active sites tend to generate CO as the main product, and those with In^41,42^ or Sn^43,44^ result in formate. Since the copper center has a negative adsorption energy for an essential intermediate *CO and a positive adsorption energy for *H,^45,46^ Cu-based catalysts show an enormous advantage in electrochemical reduction of CO_2_ to the high value-added products, such as hydrocarbons and carbon oxygenates (*i*.*e.*, the further reduced products), undergoing more than two-electron transfer processes.^45^ Actually, except for Cu-based catalysts, only a few catalysts with Zn(II)^47^ and Ni(II)^48^ sites can promote the generation of CH_4_ and C_3_ to C_6_ products, respectively. Thus, Cu-based MOF and relevant catalysts are the main focus of this article, and the common reaction pathways and key intermediates involved in the literature on MOF electrocatalysts are summarized in Figure 3.

**Table 1:** Comparison of FEs (%) for Yielding CH_4_, C_2_H_4_, and C_2+_ Products by Using Different Cu-MOFs for ECR

product	CH_4_	C_2_H_4_	C_2+_	ref.
**Cu^II^_4_**-**MFU-4*l***	92	0	0	(22)
**CuDBC**	80	∼5	∼5	(25)
**NNU-33(H)**	82	∼5	∼5	(26)
**NNU-50**	66.4	∼15	∼15	(27)
**MAF-2E**	20	51.2	51.2	(21)
**PcCu-Cu-O**	15	50	50	(28)
**Cu**-**HITP@PDA**	3	50	75	(29)
**Cutrz**	3	50	80	(23)

### Design of Active Site Structures

**Figure 4:**
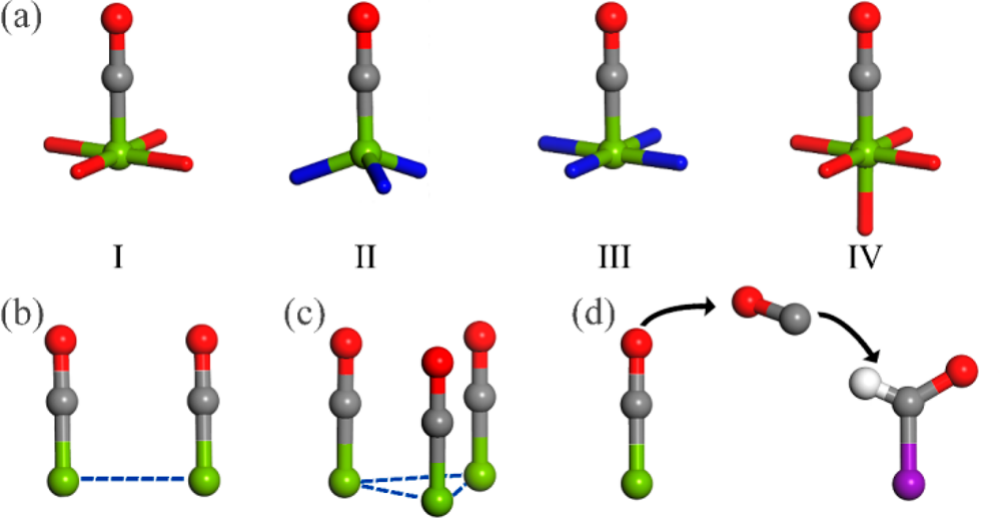
Potential relationships between Cu site structures and the behaviors of *CO intermediates. (a) Single copper sites: (I) square-planar CuO_4_; (II) trigonal pyramidal Cu(I)N_3_; (III) square-planar CuN_4_; (IV) square-pyramidal CuO_5_, (b) dicopper site, (c) tricopper site, and (d) dual copper site. Color codes: carbon (gray), copper (green and purple spheres respectively represent two types of structurally different copper sites with significantly longer adjacent Cu···Cu distances (>4 Å)), hydrogen (white), nitrogen (blue), and oxygen (red).

Obviously, the structures of active sites have a great impact on the electrochemical behaviors and ECR performances of MOFs. Different from single-atom materials and metal nanoparticles, the designable coordination structures endow MOFs with a diversity of definite active sites.^5^ Typically, for Cu-based MOFs, the structures of active sites can affect the product selectivity by influencing the possibility of C–C coupling between C_1_ intermediates. Ordinarily, as shown in Figures 3 and 4a, the discrete metal center might play a role as a single active site in ECR, leading to C_1_ compounds (and under certain circumstances, acetate) as the main products. In most cases, the production of CO, CH_4_, and acetate shares the *CO intermediate in their pathways despite rare exceptions (e.g., *HCOOH instead of *CO intermediate for yielding CH_4_ according to Lan et al.^26^), which requires the infeasibility of C–C coupling. For example, the square-planar CuO_4_ sites (I in Figure 4a) in **Cu-THQ** (H_4_THQ = tetrahydroxy-1,4-quinone),^49^*in situ* generated trigonal pyramidal Cu(I)N_3_ sites (II in Figure 4a) in [Cu_4_ZnCl_4_(btdd)_3_] (**Cu**^**II**^_**4**_**-MFU-4***l*, H_2_btdd = bis(1*H*-1,2,3-triazolo-[4,5-*b*],[4′,5′-i])dibenzo-[1,4]-dioxin),^22^ and square-planar CuN_4_ sites (III in Figure 4a) in porphyrin units^50^ exhibit impressive electrochemical performances for yielding CO and/or CH_4_ in ECR because of the inhibition of C–C coupling of *CO intermediates.

Contrary to the C_1_ products, generating C_2+_ products from ECR requires the C–C coupling process of two C_1_ intermediates. Generally, the pathways of C–C coupling are largely dependent on the types of catalytic systems. For instance, as for Cu(100)^51^ and Cu(111)^52^ facets, there is an array arrangement of closely adjacent copper atoms (<2.6 Å) on their surface. Such a short Cu–Cu distance is beneficial to the direct C–C coupling between two *CO intermediates (*i*.*e.*, the *CO–*CO coupling to yield *OCCO). In MOFs, di- and tricopper sites (Figure 4b,c) usually take the form of either *CO–*CHO or *CO–*COH coupling (Figure 3c,d) instead of the *CO–*CO coupling since they have di- or multiple metal sites with adjacent Cu···Cu separations of ∼3.3 to ∼3.6 Å.^23,53,54^ Lan’s group reported that a series of one-dimensional (1D) coordination polymers, **Cu-PzX** (X = H, Cl, Br, I), with dicopper sites (Figure 4b) can allow the C–C coupling of *CO–*COH to yield C_2_H_4_.^54^ Besides, dicopper sites in **CuBtz**(55) and **MAF-2E**(21) have also been reported, which led to C_2+_ compounds as the main products and will be discussed in subsequent sections. We further revealed by periodic density functional theory (PDFT) calculations that a 3D MOF, [Cu_3_(μ_3_–OH)(μ_3_-trz)_3_(OH)_2_(H_2_O)_4_]·*x*H_2_O (**Cutrz**, Htrz = 1*H*,1,2,4-triazole),^23^ can bind three C_1_ intermediates at its tricopper active site prior the formation of *CO. The three reduced *CO intermediates can be aligned in a parallel fashion on the same side (schematically depicted in Figure 4c), achieving a higher *CO coverage to further promote the coupling of *CO and its hydrogenated *COH intermediate, thus leading to a better FE(C_2+_) of >80%.

Apart from the adjacent di- and tricopper sites, the dual copper site (Figure 4d) has also been developed for yielding C_2+_ products. There are two types of structurally different copper sites with significantly longer adjacent Cu···Cu distances (>4 Å) in the dual copper sites; hence, the migration of CO species for the C–C coupling is necessary. In other words, the dual copper site systems feature a tandem pathway for the generation of CO species and subsequent C–C coupling. We recently constructed two electrocatalytic systems with dual sites, namely, **PcCu-Cu-O** (with both CuO_4_ and CuPc sites, the adjacent Cu···Cu is separated by 8.95 Å, CuPc = copper-phthalocyanine)^28^ and **Cu(111)@Cu-THQ** (with CuO_4_ and Cu(111) sites).^52^ Since the square-planar CuO_4_ site always shows a high selectivity for yielding CO (more analyses will be given in the subsequent section), it can serve as a CO source, and the CO species can migrate to couple with a *CHO intermediate generated at an adjacent CuPc or Cu(100) site, which has a stronger binding and reduction ability to CO and hence facilitates the formation of *CHO intermediate and the subsequent C–C coupling. Therefore, the tandem pathway on dual copper sites can also result in an excellent C_2+_ selectivity. Different with the dual copper site, in a **PcCu-TFPN** covalent-organic framework (COF) with identical isolated copper sites for ECR, the active site allows the generation of a *CH_3_ intermediate and the asymmetrical C–C coupling between a *CH_3_ species and a CO_2_ molecule, resulting in the formation of acetate.^56^ The detailed mechanism will be discussed in the next section. These facts indicate that controlling the hydrogenation of *CO intermediates and subsequent C–C coupling of *CO–*CHO or *CO–*COH are of great importance for tuning the MOF-catalyzed ECR selectivity toward C_1_/C_2_ products, and MOFs with two or more closely located metal sites are conducive for the C–C coupling to yield C_2_ or C_2+_ products. The potential relationships are intuitively illustrated in Figure 4, excluding the active site for yielding formate because it is mostly irrelevant to the *CO intermediate.

### Control of Electron Property of Active Site

The selectivity of different C_1_ products largely depends on the electron structure of the active site. MOFs and COFs with Co(II)^33,34^ or Ni(II)^36−38^ single active sites tend to generate CO as the main product. In contrast, the Cu single sites may lead to a variety of products. Many investigations have demonstrated that a copper active site with a relatively low valence and high charge density can form a strong interaction with a *CO intermediate, thereby promoting the generation of further reduction products.

**Figure 5:**
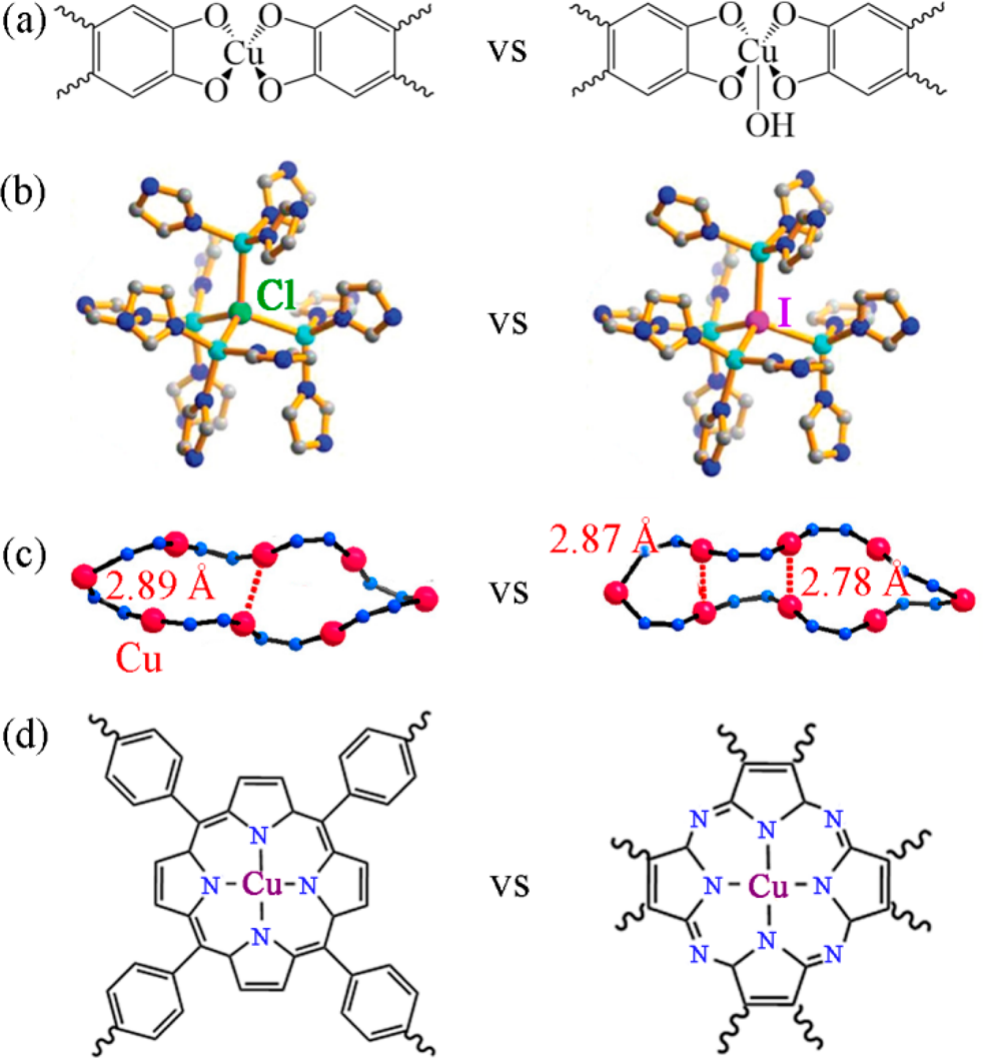
Some reported strategies to regulate the charge density or *d-*orbital energy levels of active sites. (a) Optimization of coordination geometry, (b) Decreasing the electronegativity of a coordinated atom, (c) enhancement of cuprophilic interaction, and (d) intensification of an electron-donating effect. Adapted with permission from refs (26, 57, and 59). Copyright 2021 and 2022 American Chemical Society, and from ref (56). Copyright 2022 Wiley-VCH GmbH.

The square-planar CuO_4_ site has weak affinity for CO; i.e., the *CO intermediates tend to desorb to form CO molecules instead of subsequent hydrogenation into hydrocarbons.^22,57^ For example, both **Cu-THQ**(49) and **Cu-HHTT** (H_6_HHTT = 9,10-dihydro-9,10-[1,2]benzenoanthracene-2,3,6,7,14,15-hexaol)^58^ with CuO_4_ sites exhibit high selectivity for yielding CO. Generally, the binding strength between the Cu site and CO species is highly dependent on the local charge, electronic distribution, and *d*-orbital energy levels of the Cu active site.^57^ Therefore, some strategies to adjust the energy levels of electrons in the *d*-orbitals (or *d*-band) by the coordination geometry (or coordination field) can tune the stability of *CO intermediate on the active site. For instance, 3D MOF **Cu-DBC** (H_8_DBC = dibenzo-[*g*,*p*]chrysene-2,3,6,7,10,11,14,15-octaol) with square-pyramidal CuO_5_ sites (IV in Figure 4a)^25,57^ was reported for ECR to produce CH_4_ with FEs of 56% and 80% in 0.1 M KHCO_3_ and 1 M KOH electrolytes, respectively, thanks to the change of *d*-orbital energy levels. Compared with the square-planar CuO_4_ site, the CuO_5_ site with an additional axial oxygen atom leads to energy level elevations of the *d*_*z*^2^_, *d*_*xz*_, and *d*_*yz*_ orbitals, thus enhancing the Lewis basicity of the Cu site and boosting the electron donation from the Cu center to the empty π* orbital of CO species. Therefore, the CO species can bind tightly on the CuO_5_ site to form a more stable *CO intermediate (binding energy of −73.4 kJ mol^–1^ for the CuO_5_ site versus that of −48.6 kJ mol^–1^ for the CuO_4_ site in **Cu-DBC**) (Figure 5a), as being verified by PDFT calculations, which promotes further hydrogenation of *CO to yield CH_4_.^57^ Similarly, Sun et al. designed a series of MOFs with Cu_4_X (X = Cl, Br, or I) clusters (denoted as **Cu–Cl**, **Cu–Br**, and **Cu–I**, respectively) (Figure 5b) and elaborated the effect of halogen ligand on the electron structure of Cu site for ECR.^59^ According to the DFT results, with the increasing radius of the halogen atom from Cl to I, the *d-*band center of the Cu site positively shifts to the Fermi level, and the formation energies of the key intermediates *CH_2_O and *CH_3_O were successively reduced, leading to an enhanced Faradaic efficiency of CH_4_. Apart from the design of ligands, metal–metal interactions also have an impact on the electron structure of the metal ions. Lan et al. reported an **NNU-33(H)** MOF as an ECR catalyst with adjacent Cu(I) ions (separated at 2.81 Å), in which the C–C coupling might be suppressed by the steric hindrance, resulting in a low selectivity for yielding C_2+_ products. Instead, **NNU-33(H)** exhibits a very high selectivity for electroreduction of CO_2_ to CH_4_ because of the inherent intramolecular cuprophilic interactions between two Cu(I) ions (Figure 5c).^26^ Briefly, one of the two Cu(I) ions shows an electron donating behavior toward another one, which efficiently enhances the charge density of the latter Cu(I) site. Compared with **NNU-32** without significant Cu–Cu interaction, **NNU-33(H)** exhibits an enhanced performance for the electroreduction with an FE(CH_4_) of 82% (vs 53%) (Figure 5). A similar cuprophilic interaction also exists in an another MOF **NNU-50**, which exhibits an FE(CH_4_) of 66.4%.^27^ All the obvious differences in product selectivity of the aforementioned electrocatalysts are mainly attributed to the differences in electronic distribution and orbital energy levels of Cu sites.

Unpredictably, as an isolated active site with a more enhanced Lewis basicity, the Cu-phthalocyanine site in a COF **PcCu-TFPN** (Figure 5d) exhibits a high selectivity for the C_2_ product acetate in ECR.^56^ Compared with a classical single-atom copper catalyst (**CuSAC**) as well as a copper-porphyrin-based COF (**Cu-porphyrin**), **PcCu-TFPN** has a stronger electron delocalization and higher electron density around its Cu active sites, which should be attributed to the high abundance of electron-rich nitrogen atoms in the phthalocyanine units. Therefore, unlike **CuSAC**, which generates CO as the main product, **PcCu-TFPN** can form a stronger interaction with *CO species, thus suppressing release of CO as the product. Furthermore, although both **Cu-porphyrin** and **PcCu-TFPN** can promote the reduction of *CO to *CH_3_ intermediate, the special electron distribution property of **PcCu-TFPN** leads to a lower oxidation state of the C atom in the *CH_3_ intermediate. Consequently, the nucleophilic *CH_3_ intermediate on **PcCu-TFPN** can adsorb a second CO_2_ molecule as a Lewis acid to achieve an asymmetric C–C coupling into an acetate (Figure 3b), while **Cu-porphyrin** results in CH_4_ as the main product (Figure 3a). Altogether, we may conclude that the enhanced Lewis basicity of a single copper active site tends to result in methane and acetate as main products. Most importantly, combining the above discussions, the conclusion of Figure 4 can be expanded to that the C–C coupling of a *CO and another C_1_ intermediate, generated from two closely adjacent active sites, tends to result in C_2+_ products (mostly C_2_H_4_ and C_2_H_5_OH) as main products. In contrast, the enhanced electron density of an isolated metal active site is conducive to the formation of further reduced C_1_ intermediates or products (e.g., *CH_3_ and CH_4_), while it inhibits the C–C coupling of a *CO with another C_1_ intermediate.

### Design of Chemical Microenvironment

Along with efficient metal sites, the reasonable design of a chemical microenvironment around the metal sites can significantly enhance ECR performances, which may be considered to be inspired by the synergistic effect of the unique coordination geometry of metal site and its microenvironment of a biological metalloenzyme to achieve exceptional catalytic activity and selectivity. Typically, one can construct the microenvironment of an active site by introducing special functional groups around the active sites in the catalytic system.^29,55,60−63^ Thanks to the tailorable structures of MOFs, regulation of the chemical microenvironment around the active sites in MOFs is highly feasible, which provides an unique opportunity for the design of proton-based interactions and control of framework flexibility, and is important to tune the catalytic performances of MOFs.

**Figure 6:**
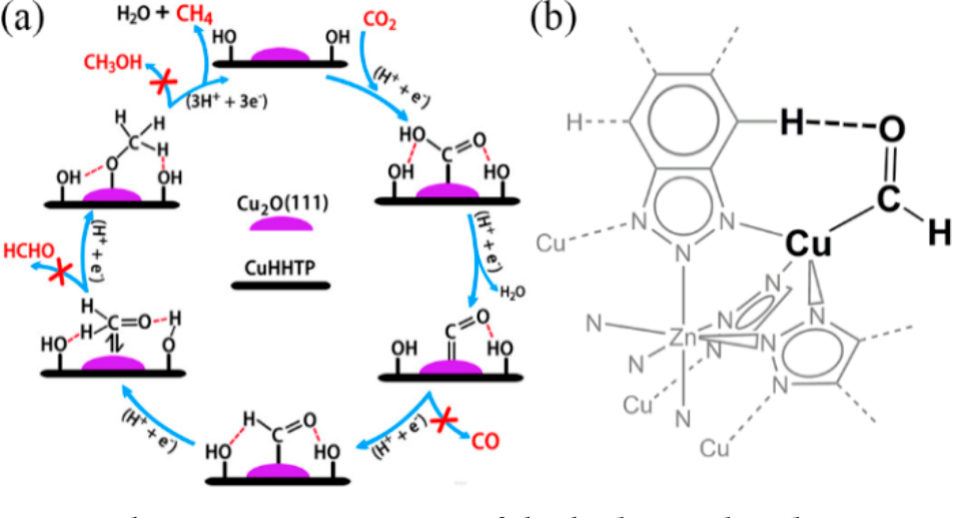
Schematic presentations of the hydrogen-bonding interactions of ECR intermediates in (a) **Cu**_**2**_**O@CuHHTP** and (b) **Cu**^**II**^_**4**_**-MFU-4***l*, respectively. Reproduced with permission from ref (65), Copyright 2020 Wiley-VCH GmbH, and from ref (22), Copyright 2021 American Chemical Society.

Different from single-atom materials and metal nanoparticles, the rational design of the secondary coordination sphere or chemical microenvironment of MOFs with proton-rich structures can allow the active sites of MOFs to establish hydrogen-bonding interactions with not only CO_2_ at the very initial step^64^ but also different intermediates in the course of ECR to enhance the binding strengths between the active sites and the substrates. For example, Cao et al. recently developed a **Cu**_**2**_**O@CuHHTP** composite system, in which **CuHHTP** has uncoordinated hydroxyl groups in H_6_HHTP ligands.^65^ Attenuated total reflection Fourier transform infrared spectroscopy (ATR-FTIR) spectra and PDFT calculations revealed that Cu_2_O(111) plane serves as active sites, where the intermediates form hydrogen bonds with the neighboring uncoordinated hydroxyl groups (Figure 6a). These hydrogen-bonding interactions efficiently assist to stabilize the ECR intermediates, which are conducive to the further reduction into CH_4_. Similarly, the aforementioned **Cu**^**II**^_**4**_**-MFU-4***l* also reveals the role of hydrogen-bonding interactions in ECR.^22^ The *CHO intermediate adsorbed on the Cu(I)N_3_ site can form a nonclassical or weak hydrogen bond with a neighboring aromatic hydrogen atom of **Cu**^**II**^_**4**_**-MFU-4***l* (Figure 6b), enhancing the intermediate’s stability. Compared with the postsynthetic composites such as **Cu**_**2**_**O@CuHHTP**, these well-defined interaction structures of MOF electrocatalysts at the atomic level show clear reduction mechanisms.

**Figure 7:**
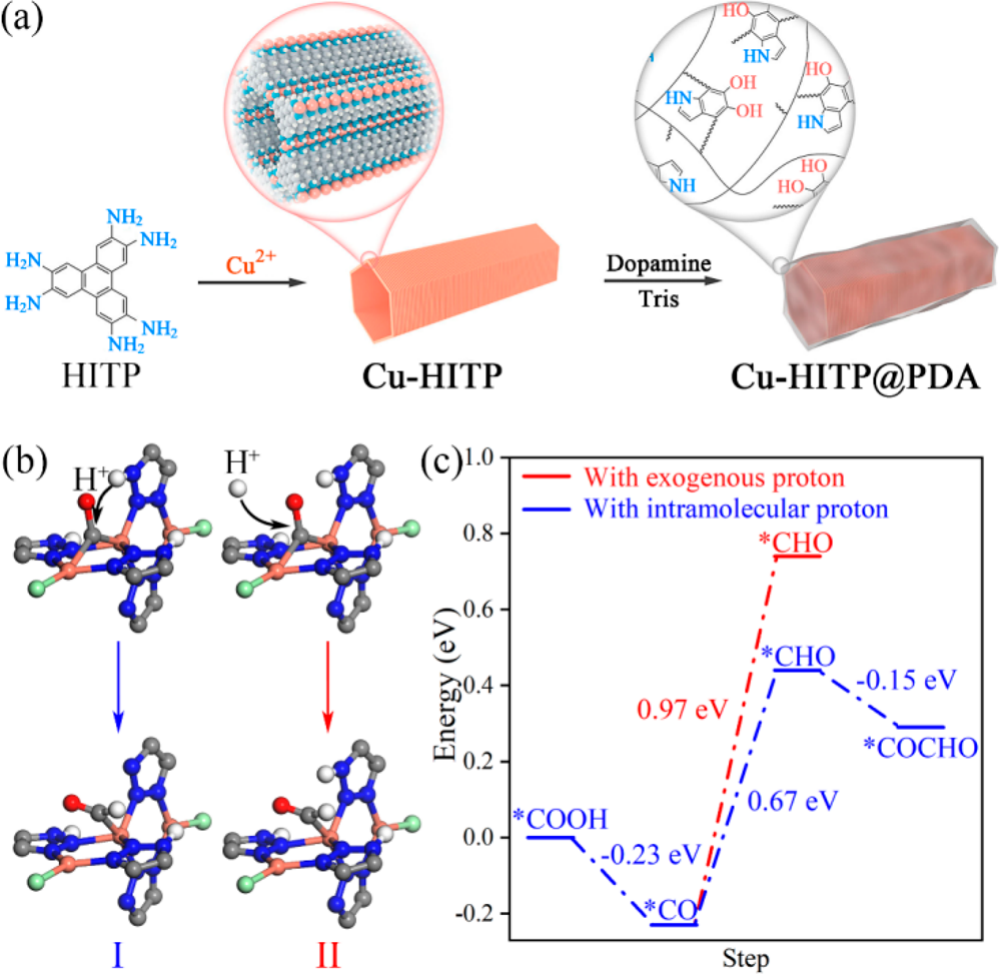
Schematic drawings for (a) the preparation of **Cu-HITP** and **Cu-HITP@PDA** illustrating the MOF structure and coated proton source/relay and (b) two possible processes (I: with intramolecular proton; II: with exogenous proton) for the hydrogenation of *CO to *CHO on **CuBtz** during ECR. Color codes: carbon (gray), chloride (light green), copper (orange), hydrogen (white), nitrogen (blue), oxygen (red). (c) The corresponding Gibbs free energy barriers of the elementary steps on **CuBtz** during the ECR pathway. Reprinted with permission from refs (29 and 55). Copyright 2022 American Chemical Society.

Apart from hydrogen-bonding interactions, proton-rich structures of MOFs or the chemical microenvironment around the active sites can also serve as proton sources (or proton donors) in ECR, which has attracted attention lately.^60,63,66−69^ When the functional groups acting as Brønsted acid sites (e.g., hydroxyl and amino groups) are located in the vicinity of the metal sites, the intermediates of CO_2_ reduction can receive protons from the adjacent Brønsted acid sites rather than directly from the electrolyte. For instance, we recently compared the performances of three polymer-coated **Cu-HITP** (a 2D MOF with square-planar CuN_4_ nodes and interlayer Cu···Cu distance of 3.4 Å) composites, namely, **Cu-HITP@PDA** (HITP = 2,3,6,7,10,11-hexaiminotriphenylene; PDA = polydopamine, with rich amino groups and phenolic hydroxyl groups as proton donors), **Cu-HITP@PANI** (PANI = polyaniline, with only amino groups), and **Cu-HITP@Poly(p-vinylphenol)** (with only phenolic hydroxyl groups) featuring different chemical microenvironments around the same catalytic sites.^29^ Compared with **Cu-HITP@PDA** (Figure 7a), both **Cu-HITP@PANI** and **Cu-HITP@Poly(p-vinylphenol)** exhibit significantly diminished electrochemical performances for C_2+_ production, attributed to much less amine or phenolic hydroxyl groups for the hydrogen-bonding interactions and proton source. More recently, we designed and prepared a porous molecular material **CuBtz** that is formed by π–π stacking interactions between discrete trinuclear [Cu_3_(HBtz)_3_(Btz)Cl_2_] clusters (HBtz = benzotriazole) into a MOF-like structure, as being characterized by powder X-ray diffraction.^55^ In the well-defined porous structure of **CuBtz**, the dicopper(I) active sites (Figure 4b, Cu···Cu distance = 3.52 Å) are closely adjacent to uncoordinated nitrogen atoms on the triazole ligands (Figure 7b). As has been evidenced by a substantial reduction of ECR performance through replacement of the triazole ligands with analogous indole ligands without uncoordinated nitrogen atoms and N–H groups into the trinuclear cluster, such uncoordinated nitrogen atoms and N–H groups on the triazole groups serve undoubtably as highly efficient proton relays, which can effectively reduce the Gibbs free energy barrier of the potential determining step, to facilitate the ECR of C_2+_ production (FE = ∼74%) (For details about the mechanism, see the following section). The above investigations demonstrate clearly that, as one kind of chemical microenvironment, the construction of appropriate proton relays is critical to improve the selectivity for yielding further reduced products in ECR.

**Figure 8:**
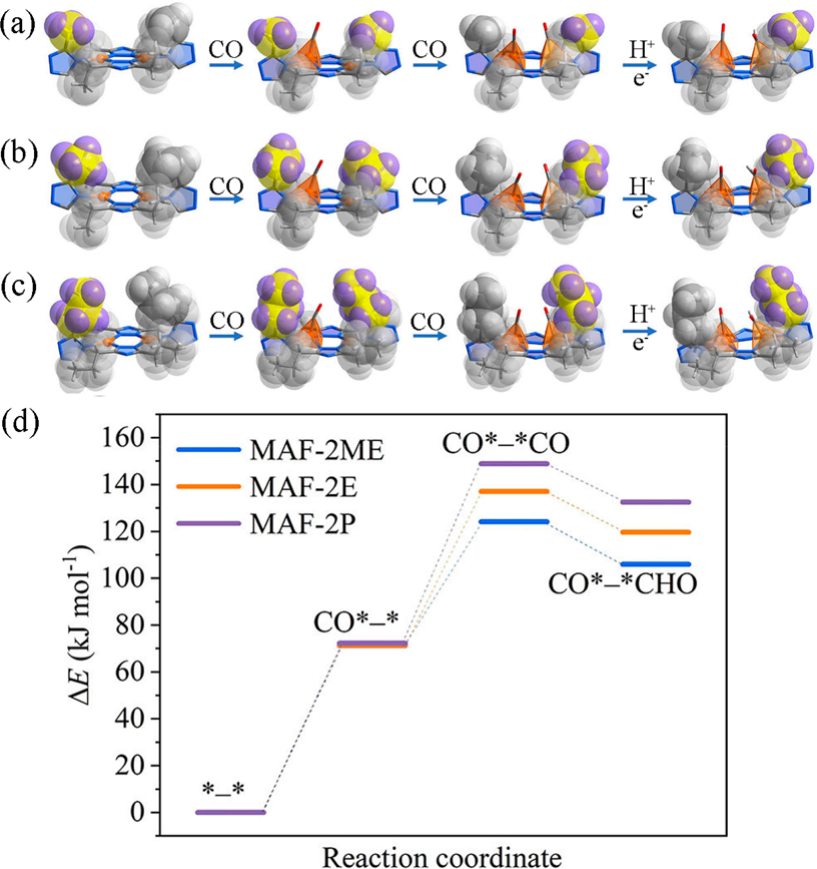
Structures of *, *CO, CO*–*CO, and CO*–*CHO intermediates for (a) **MAF-2ME**, (b) **MAF-2E**, and (c) **MAF-2P**, respectively. (d) Free energies of different intermediates binding on **MAF-2ME**, **MAF-2E**, and **MAF-2P**. Color codes: carbon (gray), copper (orange), hydrogen (white), nitrogen (blue), oxygen (red). Reproduced with permission from ref (21). Copyright 2022 Wiley-VCH GmbH.

Moreover, as a typical feature of many MOFs, the flexibility allows the controllable host–guest interaction in MOFs for adsorption and separation.^70,71^ The flexibility of MOFs can even affect the microenvironment of catalytic active sites, hence the selectivity of products as very recently documented by Zhang and co-workers that three isoreticular MAFs, [Cu(detz)] (**MAF-2** or **MAF-2E**, Hdetz = 3,5-diethyl-1,2,4-triazole), [Cu(dmtz)_0.33_(detz)_0.67_] (**MAF-2ME**, Hdmtz = 3,5-dimethyl-1,2,4-triazole), and [Cu(dptz)] (**MAF-2P**, Hdptz = 3,5-dipropyl-1,2,4-triazole) with different triazolate ligands or different ratios of triazolate ligands (Figure 8) give different selectivities of C_2_H_4_/CH_4_ in ECR.^21^ These MAFs possess dicopper sites (Figure 4b, Cu···Cu distance = 3.4 Å) exposed on the pore surfaces and have different sizes of triazolate side groups (methyl, ethyl, and propyl) in the frameworks. Very interestingly, as the size of ligand side group increases, the product ratio of C_2_H_4_/CH_4_ can be gradually tuned and even inversed from 11.8:1 to 1:2.6. PDFT simulations showed that the trigonal copper sites transform to tetrahedral upon binding the reaction intermediates, and the dicopper sites can distort accordingly to furnish the formation of C_1_ intermediates and C–C coupling. Notably, the smaller ligand side groups have less steric hindrance effect, allowing sufficient distortion for the simultaneous binding of two *CO intermediates, and subsequently one *CO and one *CHO intermediates on the dicopper site to yield C_2_H_4_ as a preferential product. In contrast, the larger ligand side groups restrict the distortion of the framework for the simultaneous binding of two intermediates on the dicopper site, leading preferentially to produce CH_4_. This work demonstrates well that the MOF flexibility can also serve as a microenvironment factor in the product selectivity of ECR.

## Current Density Improvement

**Figure 9:**
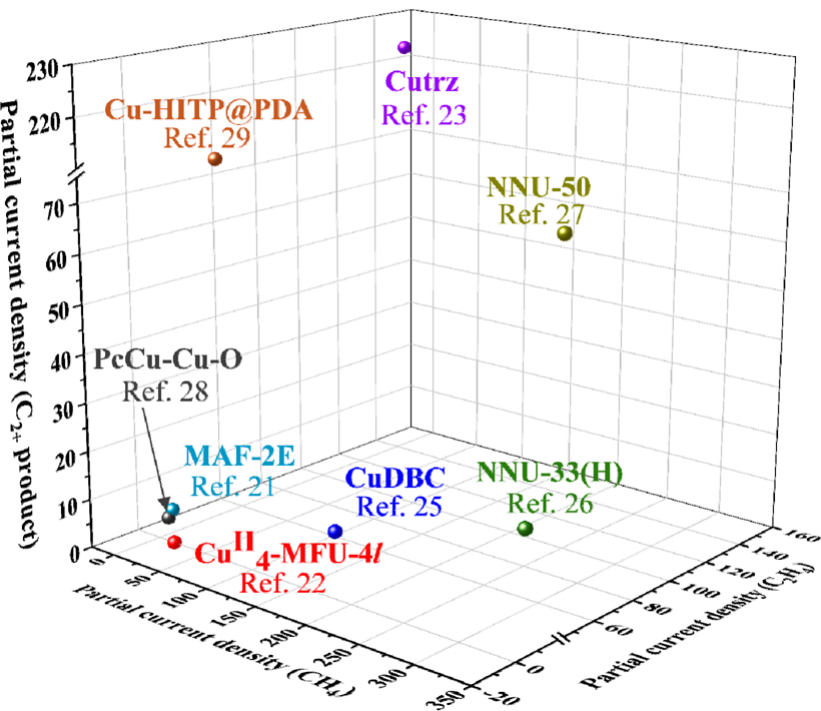
Comparison of current densities for yielding CH_4_, and C_2_H_4_, and C_2+_ products of different Cu-MOFs for ECR.

In a typical electrochemical reaction, the conversion of reaction substrate requires the participation of electrons; thus, the current density directly reflects the reaction efficiency. MOFs usually show low electric conductivity; by far the highest partial current density for CH_4_, C_2_H_4_, and C_2+_ products are just 320,^26^ 140, and 224 mA cm^–2^ in alkaline electrolyte,^23^ respectively (Figure 9 and Table 2). These current densities are far from the values (at least 360–510 mA cm^–2^) required for industrial applications.^72^ Therefore, necessary measures should be taken for the improvement of the current density of ECR.^73^

**Table 2:** Comparison of Partial Current Densities (mA cm^–2^) for Yielding CH_4_, C_2_H_4_, and C_2+_ Products by Using Different Cu-MOFs for ECR

product	CH_4_	C_2_H_4_	C_2+_	ref
**Cu**^**II**^_**4**_**-MFU-4***l*	16	0	0	(22)
**CuDBC**	162.4	∼10.2	∼10.2	(25)
**NNU-33(H)**	320.6	∼19.6	∼19.6	(26)
**NNU-50**	300	70	70	(27)
**MAF-2E**	2	5.1	5.1	(21)
**PcCu-Cu-O**	1.1	3.7	3.7	(28)
**Cu-HITP@PDA**	3	50	75	(29)
**Cutrz**	8.4	140	224	(23)

**Figure 10:**
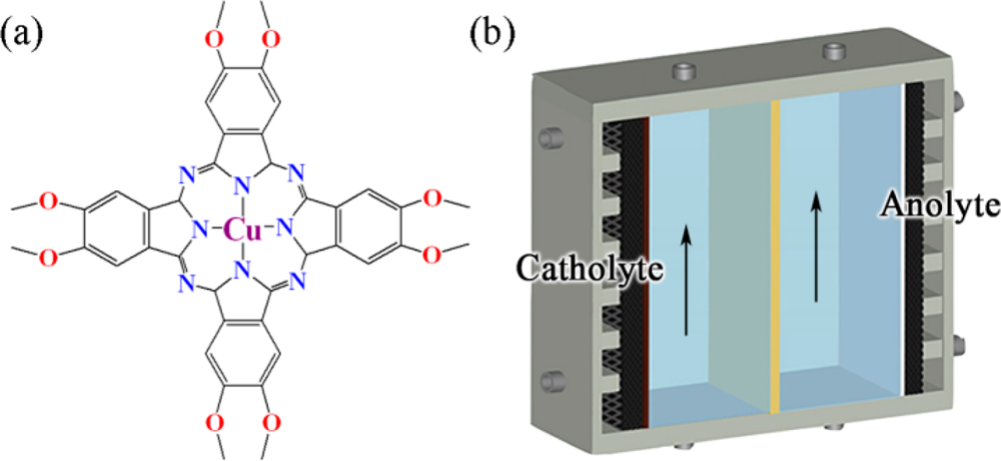
Two recently reported strategies to improve the current density of MOF catalysts for ECR. (a) The use of conductive ligands and (b) electrolyzer optimization. Adapted with permission from ref (28). Copyright 2021 American Chemical Society, and ref (79). Copyright 2021 Elsevier Inc.

In most studies, the catalyst particles were simply coated on a conductive substrate, such as gold/copper/nickel/silver foil/foam, glassy carbon (GC), indium tin oxide (ITO) glass, and fluorine-doped tin oxide (FTO), while binders such as poly(vinyl alcohol) and Nafion (sulfonated tetrafluorovinylfluoropolymer copolymer) with negligible electronic conductivity are commonly used to prevent catalyst peeling off. To enhance the electrical contact between the substrate and MOF particles, a chemically inert but conductive material such as carbon black and/or carbon nanotube can be used as an additive.^74,75^ Electrophoretic deposition of MOF onto the conductive substrate is another effective method to form good electrical contact. However, *in situ* growing the catalyst directly on the conductive substrate as a nanocrystalline film should be the better way to strengthen both electrical contact and mechanical stability.^28,58^ On the other hand, the current density can be improved by other advanced modulations, for example, (i) design of the microenvironment of active sites to reduce the kinetic energy barrier (Figure 7b); (ii) regulation on the intrinsic properties of MOFs by using large conjugated organic ligands to enhance the intrinsic electronic conductivity of MOFs (Figure 10a); (iii) optimization of the electrolyzer configuration (Figure 10b). Besides, inspired by the discussion in the previous section about the significant effect of a proton-rich microenvironment as proton relays on the performance, presumably, we may be expect that a proper polymer binder with proton-rich donors and acceptors should boost the catalytic performance.

Reduction of the kinetic energy barrier of ECR can significantly result in a high reaction rate, thus leading to high current density. Actually, some properties which can improve ECR selectivity toward a single component product mentioned in the prior section, such as *d*-orbital upshift, electron density enhancement of the metal site, and proton source configuration, might also lead to a diminished activation energy of the rate-determining step. In the case of **CuBtz**,^55^ the uncoordinated nitrogen atoms and N–H groups can serve as highly efficient proton relays for promoting the transfer of dissociated protons from the electrolyte to ECR intermediates (Figure 7b), thus accelerating the proton transfer process and reducing the reaction energy barrier of the key step of C–C coupling. Consequently, **CuBtz** exhibited a very high current density of ∼1 A cm^–2^ in 1 M KOH solution. Therefore, the secondary coordination sphere or the chemical microenvironment of MOF catalysts should be rationally designed to achieve an optimal dynamic process and higher current density.

Conductive ligand design has an essential impact on the intrinsic conductivity of MOFs. The organic ligands with large π-conjugated structures can effectively manipulate the electron transfer ability of MOFs.^76,77^ Mirica et al. developed a series of 2D, π-conjugated phthalocyanine MOFs with different metal ions.^38^ Thereinto, **CoPc-Cu-O** exhibits a conductivity of 2.12 S m^–1^ and a current density of 9.5 mA cm^–2^ with an FE(CO) of 79% in 0.1 M KHCO_3_ solution. Similarly, the aforementioned **PcCu-Cu-O** (Figure 10a) exhibits a high conductivity of 5.0 S m^–1^ and thus shows an appreciable current density of 7.3 mA cm^–2^ in 0.1 M KHCO_3_ electrolyte.^28^ Therefore, the phthalocyanine MOFs usually show high conductivity and are suitable for electrochemical applications thanks to their large conjugated structures. Inspired by the nitrogen atom configuration in phthalocyanine based MOFs, we note that the incorporation of nitrogen-rich structures into some other 2D MOFs can also improve the current density of ECR. Chen et al. compared the structures and electrochemical performances of **Cu**_**3**_**(HHTQ)**_**2**_ (HHTQ = 2,3,7,8,12,13-hexahydroxytricycloquinazoline) and **CuHHTP**, where the former has a N-rich conjugated configuration, and the latter merely has a triphenylene structure.^78^ As a result, **Cu**_**3**_**(HHTQ)**_**2**_ shows a higher current density of 45 mA cm^–2^ at a potential of −1.2 vs a reversible hydrogen electrode (RHE) than that of **CuHHTP** (30 mA cm^–2^). These results demonstrate that the N-rich conjugated ligands in MOFs can be significantly conducive to the improvement of current density, which sheds light on designing novel ligands with a higher electron transfer ability for ECR.

**Figure 11:**
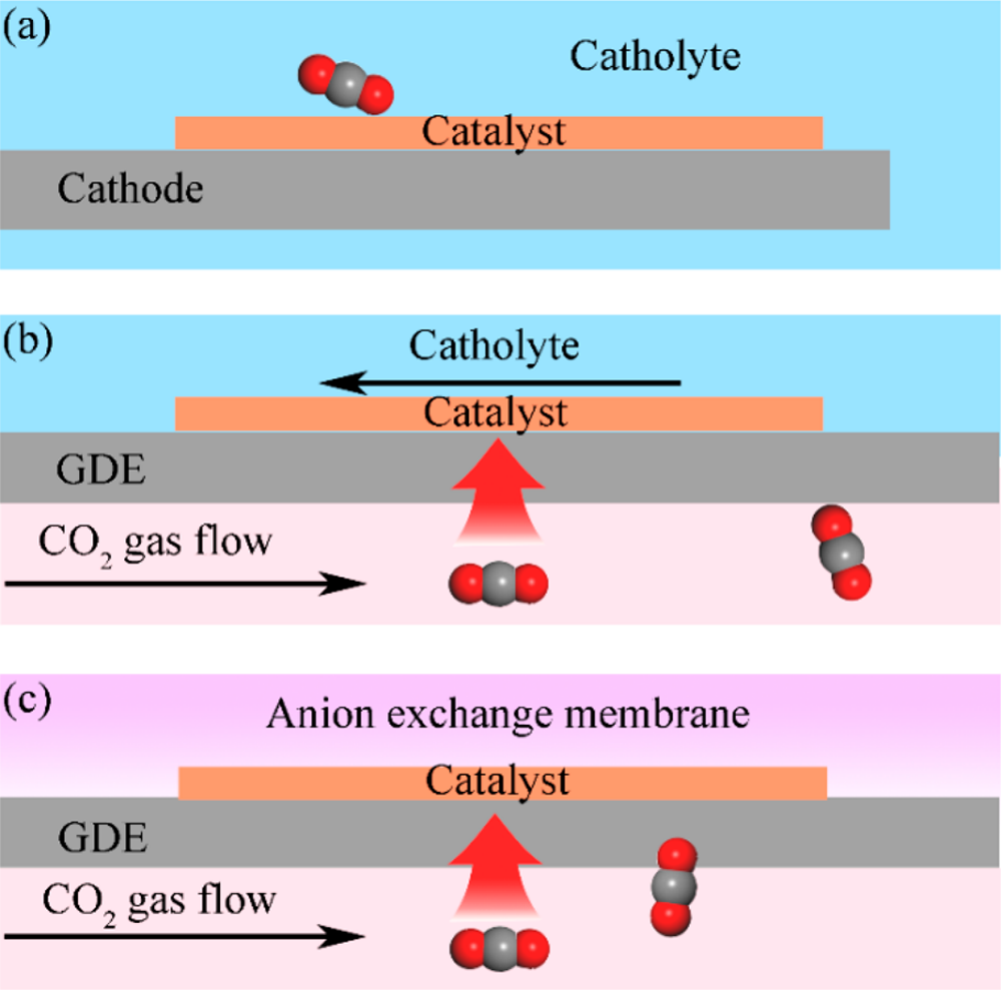
Illustration of the cathodes in (a) H-type cell, (b) liquid-phase flow cell, and (c) MEA equipped liquid-free flow cell.

Selection and optimization of electrolysis device can absolutely provoke the improvement of catalytic efficiency. It is widely accepted that the heterogeneous ECR reaction usually requires a complex three-phase interface of CO_2_ gas-electrolyte-electrocatalyst. Therefore, different manipulations of the three-phase interface breed diverse electrolysis devices. Two types of electrochemical cells, namely, H-type cell and liquid-phase flow cell (Figure 11b), have been frequently employed for ECR.^79^ Although an H-type cell is easy for assembly, it has fatal disadvantages: (i) Due to the bad CO_2_ gas contact with the catalyst on the three-phase interface, the current density is always limited; (ii) it is unsuitable for an alkaline catholyte since CO_2_ gas directly flows into the electrolyte and may result in neutralization of the catholyte (Figure 11a). In sharp contrast, a liquid-phase flow cell is suitable for alkaline electrolyte owing to the isolation of CO_2_ gas from the electrolyte. CO_2_ molecules can penetrate the gas diffusion electrode (GDE) and reach the catholyte (Figure 11b), which allows the catalyst surface to be fully exposed to CO_2_ gas and thus significantly improves the current density. A proper device should be selected for the overall consideration of application scenarios and requirements, which will also be mentioned in a subsequent section.

## Stability Enhancement of MOFs

Apart from selectivity and current density, reaction durability is also important for ECR performance assessment, and particularly, for practical application.^1^ There are many factors that cause instability of the electrocatalysis system during the long-term electrolysis process, mainly including carbonate deposition, electrolyte flooding, as well as the chemical and mechanical stability of the MOF catalysts. Optimizing the structure and electrode of electrolytic cell,^79−81^ and using acid electrolyte^82−84^ have been considered as effective methods to solve the problems of carbonate deposition and electrolyte flooding. The electrochemical stability of a MOF is related to the strength of coordination bonds and stability of organic ligands.^73,85^ Up to now, one of the most popular preferences of the device configuration for ECR is to employ an alkaline (*e*.*g.*, 1 M KOH solution) catholyte in a liquid-phase flow cell to provoke electrocatalytic activity. Nevertheless, the high pH environment might cause the collapse of the frameworks of MOFs.^79^ According to the hard-soft-acid-base theory, the combination of high-valence metal ions (hard acids) with carboxylate ligands (hard bases) or low-valence metal ions (soft acids) with azolate ligands (soft bases) is beneficial to obtain highly stable MOFs.^10,86^ Therefore, some MAFs with Cu(I) or Cu(II) ions and pyrazolate-type ligands should be good candidates for ECR in alkaline electrolytes.^87^

Apart from chemical stability, mechanical stability of MOF electrocatalysts (*i*.*e*., the binding strength of the catalyst to the electrode) is usually ignored when designing ECR catalysts. Up to now, various electrode fabrication methods have been developed,^88^ such as drop-casting,^89^ spray coating,^41^ and vacuum filtration,^90^ most of which are postsynthetic treatments with polymer binders. The electrodes fabricated by these methods might exhibit poor mechanical stability because the catalysts might peel off owing to the disturbance of the flowing electrolytes and/or the product gas bubbles generated from the catalysts. As for MOF electrocatalysts, *in*-*situ* growth of MOFs on the electrode support (*e*.*g.*, metal foils or foams) can deal well with the above issue. For example, electrochemical synthesis of MOFs on Cu and In support can provide a strong binding force between the MOFs and supports.^42,91−93^ Other methods for MOF *in situ* growth, such as solvothermal deposition^94^ and atomic layer deposition,^95^ can also be employed to fabricate the electrode, although they are rarely reported. Another alternative is to use membrane electrode assembly (MEA) equipped liquid-free flow cell as the electrolyzer for ECR. In a typical procedure of MEA, the catalyst loaded on GDE directly contacts the anion exchange membrane (Figure 11c), and sulfuric acid solution and water circulate through an anode chamber and solid-state electrolyte chamber, respectively.^96−100^ This method can conduct ECR with high efficiency without the use of a liquid catholyte. Until now, only a MIL-68(In)-NH_2_ MOF has been employed as the electrocatalyst in MEA,^41^ which may be worthy of trying. In other words, although considerable attention has been paid to the development of highly stable MOFs, more efforts are required for further enhancing the durability of MOF electrocatalysts and electrolysis devices to satisfy the requirements of industrial applications.^73^ Altogether, the control of the electrochemical durability of MOF catalysts should focus on device construction, electrolyte environment, electrode decoration, and the intrinsic properties of MOFs.

## Summary and Outlook

In this Outlook, we concisely and systematically summarized the very recent advances of MOFs as ECR catalysts and elaborated on the critical impacts of single and multiple metal sites, metal coordination geometry, the electron structure of active site, the secondary coordination sphere or microenvironment, the strength of coordination bonds and stability of organic ligands, ligand conductivity, as well as the electrolytes on ECR performance. We also propose some critical and forward-looking insights into the microstructure design of MOF electrocatalysts for performance improvement. The electrode fabrication and electrolyzer design are also highlighted for MOF catalysts. In short, on one hand, considering their tremendous potential for ECR with respect to their merit of tunable structures, MOF catalysts can be regarded as an ideal platform for precise molecular design and exhaustive mechanism investigations, and as promising candidates for CO_2_ utilization and electrochemical production of fuels and value-added chemicals. On the other hand, MOF electrocatalysts and electrocatalytic devices still suffer from critical challenges, such as low current density and stability/durability, while other aspects should also be addressed.

Although the past decade has witnessed the rapid progress of MOF catalysts for ECR, further investigations are still needed for the systematic and thorough comprehension of the MOF-boosting ECR mechanism, including the activation of the CO_2_ molecule, and formation and transformation of intermediates. Most of the studies were based on self-consistency, in which the possible reaction pathways were first proposed and then verified by FTIR,^101,102^ Raman,^103^ and/or X-ray absorption spectroscopy^15,89,90^ characterization, and theoretical calculations. In other words, the identification of ECR intermediates was mostly based on inference rather than direct evidence. Therefore, more operando characterization methods should be developed to capture the more accurate structural information on ECR intermediates. For example, differential electrochemical mass spectrometry (DEMS) is a burgeoning method to semiquantify the products in real time, which can clarify the potential conversion of intermediates.^104,105^ This *in situ* technology should be helpful to confirm the ECR mechanisms of MOF catalysts in the future. In addition, rational design of non-Cu metal sites in MOF structures to achieve high selectivity of further reduction products should also facilitate understanding of the mechanism of electrocatalytic reduction of CO_2_ to high-value hydrocarbons and oxygenates, for which machine learning based on PDFT calculations may be helpful.

The evaluation of ECR performance was mostly based on a three-electrode system (including a cathode, an anode, and a reference electrode). Actually, the full cell configuration equipped with only a cathode and an anode is more suitable for industrial manufacture; thus, the full cell voltage should be used for the assessment of energy efficiency. To achieve high electrical energy effectiveness, the anode reaction should be well designed to reduce the full cell voltage. As a traditional anodic reaction in the ECR system, the oxygen evolution reaction (OER) usually causes terrible wastage of energy because of the poor OER performances of the most commonly used platinum and graphene anodes. Anode electrocatalysts with high OER performances should be employed for the two-electrode system for ECR evaluation. Alternatively, anodic organic reactions (e.g., methanol oxidation^106^ and octylamine oxidation^107^) can be used to couple with ECR and form a full cell.^108^ This strategy makes use of the most of electrical energy and hence is beneficial for the achievement of sustainable development and green chemistry.

In a typically industrial environment, such as coal combustion, the CO_2_ content of flue gas is about 10–15%. If the electrocatalysts could work in the diluted CO_2_ as a CO_2_ source, the cost of purification and separation of CO_2_ can be largely reduced. As MOFs have been proven to have excellent performance for capturing CO_2_ by dipole–dipole interaction, weak coordination interaction, and chemisorption, integration of high CO_2_ capture and catalytic functions into the MOFs should provide an opportunity to achieve an efficient ECR in flue gas in the future.

In summary, more efforts should be devoted to design MOF catalysts with high stability and electronic conductivity besides high activity and selectivity, as well as to develop efficient electrolytic devices and their rational integration suitable for MOF catalysts, thereby achieving highly efficient, continuous, and low cost production of ECR for industrial applications.
